# Comparative study of diagnostic efficacy of sputum and bronchoalveolar lavage fluid specimens in community-acquired pneumonia children treated with fiberoptic bronchoscopy

**DOI:** 10.1186/s12879-023-08522-3

**Published:** 2023-08-29

**Authors:** Le Wang, Sukun Lu, Yinghui Guo, Jianhua Liu, Peng Wu, Shuo Yang

**Affiliations:** Institute of Pediatric Research, Children’s Hospital of Hebei Province, 133 Zhonghua South Street, Shijiazhuang, 050031 Hebei Province China

**Keywords:** CAP, BALF, Sputum, Pediatric

## Abstract

**Background:**

Community-acquired pneumonia (CAP) is usually diagnosed in children, and the type of respiratory specimen is critical. Differences in pathogens detection between induced sputum (IS) and bronchoalveolar lavage fluid (BALF) have not been evaluated.

**Methods:**

In 2018, paired sputum and BALF samples from CAP hospitalised children with indications for bronchoalveolar lavage (BAL) were subjected to multiplex PCR for the detection of 11 common respiratory pathogens.

**Results:**

A total of 142 children with paired sputum and BALF were tested. The overall positivity rate was 85.9% (122/142) for sputum and 80.3% (114/142) for BALF. The two specimens presented almost perfect agreement between the detection on *M. pneumoniae*, influenza A, influenza B, bocavirus and RSV. In contrast, adenovirus had the lowest kappa value of 0.156, and a false negative rate (FNR) of 66.7%. Rhinovirus had the highest false positive rate (FPR) as 18.5%. The consistent rate was significantly higher in school-age children than those under 1 year old (*p* = .005). Bacterial co-infection in BALF specimens were observed in 14.8% (21/142). Of the 11 discordant pairs of specimens, 9 cases were sputum(+)/BALF(-) with adenovirus predominating.

**Conclusion:**

Our findings suggest that the consistency of results between sputum and BALF is pathogen specific. Careful consideration needs to be given to whether sputum can be used as a substitute for BALF when children are young or co-infections with bacteria are suspected.

## Introduction

Community-acquired pneumonia (CAP) remains a major cause of childhood morbidity and mortality worldwide [1]. Its clinical microbiology has considerably changed because molecular methods allow the detection of a wide range of pathogens directly from respiratory specimens with high sensitivity [2]. Therefore, the choice of sample type and sampling method is critical for enhancing the predictive value of these molecular methods [3].

Owing to the convenience of specimen collection, the diagnosis of most respiratory pathogens is performed using aspirated sputum or even upper respiratory secretions such as nasal and pharyngeal swabs [4]. Data show that these specimens are susceptible to oral colonisation, making it difficult to determine whether sputum isolates indicate infection, colonisation, or contamination [4]. Fiberoptic bronchoscopy (FOB) with bronchoalveolar lavage (BAL) is now considered an important tool for the diagnosis and treatment of pneumonia. Although BAL fluid (BALF) is a reliable specimen for the identification of lower respiratory tract infection pathogens, identification via bronchoscopy has proven challenging in children owing to the need for anaesthesia and specialised procedural expertise [5]. Few studies have compared the differences in bacterial detection between sputum and BALF; however, studies comparing the two specimens in detecting viruses as well as atypical bacteria are scarce [5, 6]. According to the Infectious Diseases Society of America and the American Thoracic Society, viruses and atypical bacteria (e.g., *Mycoplasma pneumoniae*) account for a large proportion of CAP pathogenesis in children [7]. Therefore, it is important to assess the prevalence of a wide range of pathogens in relatively accessible sputum specimens compared with alveolar lavage fluid specimens.

To the best of our knowledge, there are no reports describing the differences in the simultaneous detection of several viruses and atypical bacteria by multiplex polymerase chain reaction (PCR) between induced sputum with BALF specimens. Here, we compare the identification pf these two types of specimens for nine viruses and two atypical bacteria in children with CAP receiving BAL. This approach enables more rigorous assessment of the molecular results of different respiratory specimens in the detection of multiple pathogens.

## Methods

### Study population

Children with severe CAP or other indications for BAL hospitalised between January and December 2018 were included in this study. The diagnosis of CAP is based on evidence-based guidelines published by the World Health Organization [8]. Matched sputum and BALF specimens were collected from children treated with BAL. Patients eligible for BAL were those with persistent radiologically confirmed infiltrates, lesions, consolidation, refractory respiratory symptoms, atelectasis, bronchiectasis, or bronchial foreign bodies. BALF was collected from the No. 2 Respiratory Department according to the Chinese Pediatric Flexible Bronchoscopy Guidelines (2018 edition) [9]. The exclusion criteria were as follows: (i) children with contraindications for BAL or other chronic diseases; (ii) parents or guardians refusing BAL treatment; and (iii) sputum and BALF samples were collected more than 72 h apart.

Severe CAP was defined according to the American Thoracic Society guidelines for the CAP management [10]. Cases with persistent fever for more than 7 days and/or worsening radiological findings despite appropriate management, and wherein other pathogens were excluded were defined as refractory Mycoplasma pneumoniae pneumonia (RMPP) [11]. Clinical and demographic data were retrieved from the electronic patient system.

### Ethics approval

The study plan was approved by the Health Research Ethics Committee of Hebei Children’s Hospital. Due to the retrospective design of the study, the requirement for informed consent were waived by ethics committee. All patient data was anonymous prior to analysis.

### Sample collection

#### Induced sputum (IS)

Approximately 30 min before sputum collection, the child was administered nebulised inhalation of 3% hypertonic saline for 10–15 min and instructed to spit out the saliva and then forcefully cough up the sputum into a delivery tube containing viral transport medium (VTM) (Hopebio Technologies, Qingdao, China). For infants and children who could not cough up sputum, a skilled nurse used a sterile negative-pressure suction catheter to stimulate the throat and induce coughing for obtaining sputum samples. Next, the samples were mixed thoroughly with VTM, and 200µL supernatant was aspirated for subsequent nucleic acid extraction.

### FOB and collection of alveolar lavage fluid

An experienced and qualified physician performed the procedure by first sedating the patient with intravenous midazolam and inserting a bronchoscope through the nose. After visualising the lesion under the scope, the end of the bronchoscope was wedged in, 35-37 °C (1–3 mL/kg) saline was injected, and the suction pressure was set at 100 mmHg and aspirated immediately after lavage. The target resorbed volume was ≥ 40% of the injected volume. After gently mixing the sample, 3 mL sample was used for bacterial culture and 200µL for nucleic acid extraction. Bacterial and fungal cultures were performed according to protocols developed in our diagnostic laboratory using BALF specimens.

### Nucleic acid extraction

A total of 3µL internal control was added to each extracted sample. Pathogenic DNA and RNA from sputum and BALF were extracted by Nucleic Acid Extraction or Purification Kit on an automated extraction workstation (Smart LabAssist-16/32) according to the manufacturer’s instructions (Health Gene Technologies, Ningbo, China).

### Pathogen detection

Pathogens were tested using the Respiratory Pathogens Multiplex Kit (Health Gene Tech., Ningbo, China), a multiplex PCR-capillary electrophoresis fragment analysis method designed to detect 11 respiratory microorganisms including Influenza A (Flu A), Influenza B (Flu B), human parainfluenza virus (HPIV), respiratory syncytial virus (RSV), rhinovirus (HRV), adenovirus (ADV), human metapneumovirus (HMPV), human bocavirus (HBoV), human coronavirus (HCoV), *Chlamydia pneumoniae* (CP) and *Mycoplasma pneumoniae* (MP). The analysis was then performed in an automated manner according to an established protocol and the data was compiled by the GeXP system software provided by Beckman Coulter [12].

### Statistical analysis

The detection yields of any microbes between two specimens were compared using the χ^2^ or Fisher’s exact test by SPSS 19.0 software (SPSS Inc., Chicago, USA). Agreement was assessed using Kappa statistics (κ value 0-0.20 slight, 0.21–0.4 fair, 0.41–0.6 moderate, 0.61–0.8 substantial and 0.81-1 almost perfect) [13]. Statistical significance was concluded if *p* < .05.

## Results

### Patients’ characteristics

**Fig. 1:**
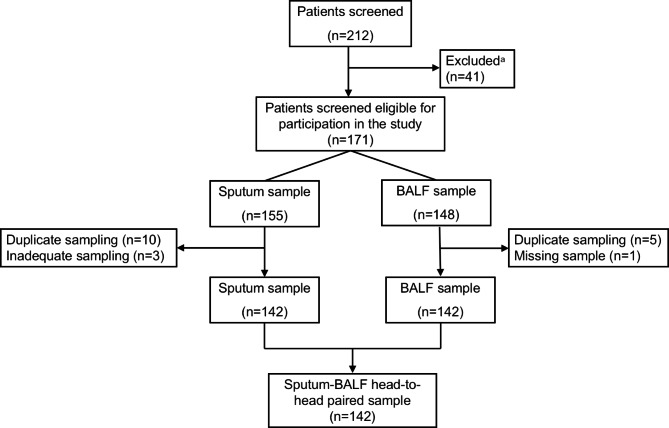
Study flowchart of patient enrollment

To compare the detection rates of sputum and BALF samples, we recruited 212 hospitalised CAP children treated with BAL between January and December 2018. Of these, cases were excluded because of the following reasons: (i) 26 samples were collected more than 72 h apart, (ii) the guardians of 15 children refused to provide paired sputum specimens, (iii) samples that were deemed insufficient for all tests. A flowchart of patient selection is shown in Fig. 1. Paired sputum and BALF samples were collected from 142 patients.

The median age of 142 paediatric patients requiring BAL was 42 months (interquartile range, 20–72 months). The male-to-female ratio was 1.33:1. Among the 142 CAP patients, severe CAP and RMPP accounting for 66.1% and 30.2%, respectively (Table 1). Using a fiberoptic bronchoscopy, the presence of bronchial foreign bodies in 5 CAP patients were observed.

**Table 1:** The diagnosis of children treated with fiberoptic bronchoscopy

Diagnosis		Number	Percentage
CAP ^a^		142	
	Severe CAP	94	66.1%
	RMPP ^b^	43	30.2%
	with Bronchial foreign body	5	3.5%

^a^ CAP, community acquired pneumonia

^b^ RMPP, refractory mycoplasma pneumoniae pneumonia

### Sputum and BALF concordance on certain pathogens

Overall, the positive rate was 85.9% (122/142) for sputum and 80.3% (114/142) for BALF. *M. pneumoniae* infection accounted for most of the enrolled cases, and its detection in sputum and BALF was in good agreement (Table 2, κ value = 0.885), and this almost perfect agreement was also observed in influenza A, B, HBoV and RSV. However, for certain organisms such as adenovirus, the concordance was slight (κ = 0.156). To better understand the inconsistent results, we assumed that the results from BALF samples were ‘gold standard’. A sample was considered ‘false positive’ if it tested as sputum(+)/BALF(−) and ‘false negative’ if it tested as sputum(−)/BALF(+). Table 2 lists the pathogens in descending order of false negative rate (FNR), showing that the FNR for *C. pneumoniae* and adenovirus were as high as 50% and 67% respectively. HRV had the highest false-positive rate (FPR) as 18.5%.

**Table 2:** Detection of 11 types of pathogens according to specimen type

Pathogens	No. of positive sputum and/or positive BALF^a^	BALF (%)	Sputum (%)	Positive concordance rate	Kappa value	False positive rate^b^	False negative rate^b^
HCoV	2	0 (0)	2 (100)	0%	-	-	-
Influenza A	10	9 (90)	10 (100)	90%	0.944	0.7%	0.0%
Influenza B	4	3 (75)	4 (100)	75%	0.854	0.7%	0.0%
HBoV	5	3 (60)	5 (100)	60%	0.743	1.4%	0.0%
*M. pneumoniae*	62	57 (92)	59 (95)	87%	0.885	5.7%	5.3%
HMPV	13	6 (46)	12 (92)	38%	0.529	5.1%	16.7%
RSV	4	4 (100)	3 (80)	75%	0.854	0.0%	25.0%
HRV	43	20 (47)	38 (88)	35%	0.410	18.5%	25.0%
HPIV	28	23 (82)	19 (68)	50%	0.610	4.1%	39.0%
*C. pneumoniae*	4	4 (100)	2 (50)	50%	0.660	0.0%	50.0%
Adenovirus	10	3 (30)	8 (80)	13%	0.156	5.0%	66.7%

^a^ Column total adds to more than the number of patients with any pathogen present, because children with more than 1 pathogen identified are also recorded

^b^ the results of BALF samples were assumed as gold standard

HRV, human rhinovirus, HPIV, human parainfluenza virus, HMPV, human metapneumovirus, RSV, respiratory syncytial virus, HBoV, human bocavirus, HCoV human coronavirus

### Sputum and OPS concordance on cases

Multiplex PCR results were consistent in 59.9% (85/142) cases, with single detections being more common than mixed detection (44.4% vs. 6.3%, Table 3). Among the discordant paired specimens, more organism types were found in sputum samples but not in BALF (26.8% vs. 9.2%). The concordance rate of pathogens in the two specimens was found to be significantly correlated with age (*p* = .005, Table 4). After Bonferroni’s multiple comparisons, the concordance rate remained significantly higher in school-age children than in children under 1 year of age (75.9% vs. 27.3%).

**Table 3:** Sputum and BALF concordance

Items			Number	Percentage
Concordant Sputum/BALF paired specimens			85	59.9%
	No pathogen detected		13	9.2%
	Single pathogen detected		63	44.4%
		*M. pneumoniae*	38	26.8%
		Influenza A	7	4.9%
		HPIV	5	3.5%
		HRV	5	3.5%
		RSV	2	1.4%
		HMPV	2	1.4%
		*C. pneumoniae*	2	1.4%
		HBoV	1	0.7%
		Influenza B	1	0.7%
	Two or three pathogens detected		9	6.3%
Discordant Sputum/BALF paired specimens			57	40.1%
	completely inconsistent		6	4.2%
	partially consistent: more types of pathogens in BALF		13	9.2%
	partially consistent: more types of pathogens in Sputum		38	26.8%

**Table 4:** Age-dependent concordance on pathogen detection in sputum and BALF.

Age (year)	No. of tested	Cases with consistent result	Consistent rate (%)	False positive rate^a^ (%)	False negative rate^a^ (%)	*p* value
< 1	11	3	27.3*	100.0	50.0	0.005
1–3	43	23	53.5	68.0	28.6	
3–5	30	15	50.0	86.7	23.5	
> 5	58	44	75.9*	76.9	8.9	

*Multiplex comparison by Bonferroni showed *p* < .05 between the two groups of < 1 and > 5 years old

^a^ the results of BALF samples were assumed as gold standard

**Table 5:** The bacterial co-infection in patients

Items					Percentage and No.
Positive bacterial detection					14.8% (21/142)
	Only bacterial detection				19% (4/21)
	Co-detection with viruses or atypical bacteria				81% (17/21)
		Consistent in Sputum and BALF			35.3% (6/17)
		Inconsistent in Sputum and BALF			64.7% (11/17)
			Sputum(+)/BALF(-)^a^		52.9% (9/17)
				Adenovirus^#92^	4
				HBoV^#92^	2
				*M. pneumoniae*	1
				HPIV^#92^	1
				HRV	1
				HMPV	1
				HCoV	1
			Sputum(-)/BALF(+)^b^		11.8% (2/17)
				Adenovirus^#13^	1
				*M. pneumoniae* ^#13^	1
				*C. pneumoniae*	1

^a^ Column total adds to more than the number of patients with Sputum(+)/BALF(-) because case #92 was co-infected with adenovirus, HPIV and HBoV.

^b^ Column total adds to more than the number of patients with Sputum(-)/BALF(+) because case #13 was co-infected with adenovirus and *M. pneumoniae*

### Bacterial co-infection

Bacterial cultures were positive in 14.8% (21/142), including *H. influenzae* (n = 11), *S. pneumoniae* (n = 8), and *S. aureus* (n = 2). Of the 21 patients with bacterial pneumonia, 81% (17/21) of them were coinfected with viruses or atypical bacteria, and no microorganisms were found in the remaining 4 BALF specimens. Of the 17 cases with co-infection, 35.3% (6/17) of the paired specimens were concordant with each other and the inconsistent result was 64.7% (11/17). Of the 11 discordant pairs of specimens, 9 cases were sputum(+)/BALF(-) with adenovirus predominating.

## Discussion

In this study, we used multiplex PCR to detect nine viruses and two atypical bacteria in paired sputum and BALF samples from 142 children hospitalised with CAP treated with BAL. There was slight, moderate to perfect agreement for these pathogens tested. As we know, sputum is the main specimen used in hospitalised patients in China due to its easy availability [14]. However, sputum specimens can be easily contaminated by pathogens present in the upper respiratory tract [15]. Alveolar lavage via fiberoptic bronchoscopy (FOB) is now a useful tool for the diagnosis and treatment of lung infections [16]. Compare to sputum, BALF specimens are much less likely to be contaminated with oral microorganisms [17]. As FOB is an invasive procedure, the possibility of using sputum rather than BALF is an issue that needs to be addressed, particularly in paediatric practice. To date, only a few reports have compared certain pathogenic results between BALF and other respiratory specimens in paediatric patients [6, 18–21].

Adenovirus infection can cause severe CAP and is associated with acute respiratory distress syndrome (ARDS) or atelectasis, with a mortality rate of over 50% in children [22, 23]. The persistence of adenovirus infection has been described as a possible cause of unremitting airway obstruction [24]. In the present study, we used multiplex-PCR analysis and the false-negative rate for adenovirus detection in sputum compared with BALF was close to 70%. Wang et al. observed a false negative rate of 58.4% for adenovirus detection by NPS compared to paired BALF in children with severe CAP [25]. These results suggest that sputum samples are occasionally not suitable for identifying the causative agent of lower respiratory tract infections, even when molecular methods are applied. Early identification of adenovirus infections from the lower respiratory tract and timely and effective treatment are important for severe CAP children in order to prevent progression of the disease.

In detecting *M. pneumoniae* nucleic acid, our previous study and others reports demonstrated the superiority of sputum over nasopharyngeal swabs (NPS) or nasopharyngeal aspirate (NPA) [20, 21, 26–28]. Luo et al. measured 533 paired NPA-BALF samples collected from children with pneumonia and found moderate concordance (κ = 0.407) for *M. pneumoniae* [21]. Xu et al. performed real-time PCR on 406 NPA and BALF samples from children with CAP and found a kappa value of only 0.020 to detect *M. pneumoniae* [20].To our knowledge, no article has compared the detection of *M. pneumoniae* between induced sputum (IS) and BALF in pediatric patients. In recent years, there has been an increasing incidence of severe Mycoplasma pneumoniae pneumonia (SMPP) and refractory MPP (RMPP) in children, and the formation of mucus plug in SMPP or RMPP is a major indication for BAL [29]. In our study, the positivity of *M. pneumoniae* was highest in children received BAL, at approximately 40%. Comparison of BALF and sputum showed almost perfect agreement, with kappa value close to 0.9, with false negative and false positive rates being approximately 5%. These data suggested that if *M. pneumoniae* has been detected in IS, repeated testing on if from BALF samples is of little significance. Similar to *M. pneumoniae*, other viruses (with the exception of adenovirus) showed good concordance. Therefore, sputum can be used as an alternative to BALF to detect *M. pneumoniae*, influenza virus, bocavirus and RSV if the purpose of patients undergoing FOB is diagnostic rather than therapeutic. Sputum can be used to detect these pathogens in children who exhibit contraindications to BAL or in children with CAP who are otherwise unable to obtain BALF.

We found that the inconsistency rate of sputum and BALF was significantly associated with age, which was higher in younger children. Rodrigues et al. observed that co-infection and carriage rates in children were independent of age [30]. Verhagen et al. found that viral co-infection was more frequent in children under 4 years of age than in older children [31]. Using a combination of clinician-ordered diagnostics and lower respiratory mNGS, Tsitsiklis et al. observed a decrease in positive detection rates with increasing age [32]. These findings can be explained by a lack of intact immune memory, reduced innate and adaptive immunity, and physiological differences in the airway, which may increase the susceptibility of children or infants to incidental carriage of potentially pathogenic microorganisms [33]. It is therefore important to select the appropriate specimen type for younger children to improve the detection accuracy on respiratory tract pathogens.

In the present study, we observed a total of 21 cases with bacterial pneumonia, of which 6 were sputum(+)/BALF(+), 9 were sputum(+)/BALF(-), 2 were sputum(-)/BALF(+) and 4 were sputum(-)/BALF(-). Of them, the proportion of inconsistent results is twice as high as the proportion of consistent results with adenovirus predominating. Ronda et al. observed increased bacterial (*S. aureus* and GNB) colonization during viral respiratory tract infections, which may be a contributing factor to the increased risk of bacterial pneumonia [34]. Du et al. showed that, 48.8% of the children (163/216) with severe adenovirus pneumonia had bacterial coinfection [35]. Lai et al. found that HMPV-infected mice showed impaired recruitment of airway neutrophils, which may lead to delayed bacterial clearance and increased inflammation in the lung [36]. Therefore, when a prior viral upper respiratory infection is suspected, it is prudent to consider whether the culprit of the pneumonia is a virus or a bacterium.

### Limitations

This study has several limitations. First, although multiplex PCR requires nominal fluorescence to determine a positive result, this method cannot be used to distinguish whether the detected pathogen is a current infection or a colonised pathogen. In addition, it is important to note that viruses take longer to shed in the upper respiratory tract than in the lower respiratory tract [37]. Future comparative studies that include the viral load measurement in a large sample size is needed. Secondly, eight patients in this study were positive for adenovirus in sputum, whereas only three patients were positive for adenovirus in BALF. Future comparative studies are needed to specifically address the differences in adenovirus detection. Third, although we kept the resorbed volume above 40% of the injected volume, the dilution of BALF may lead to missed detection of low-load pathogens. Forth, most CAP patients do not require bronchoscopy, and our comparative results were limited to patients with severe CAP, not mostly encountered CAP. Finally, although we tested most of the known pathogens causing respiratory symptoms, we cannot exclude the possibility that variants or unknown pathogens were missed.

## Conclusions

We examined, for the first time, the difference in positivity rates between sputum and BALF samples from CAP children who received BAL treatment with over a broader range of pathogens. The accordance varied across microorganisms.

## Data Availability

The datasets used and/or analyzed during the current study are available from the corresponding author on reasonable request.

## Abbreviations

BALF: Bronchoalveolar lavage fluid
CAP: Community-acquired pneumonia
IS: Induced sputum
LRTI: Lower respiratory tract infection
FOB: Fiberoptic bronchoscopy
SMPP: Severe mycoplasma pneumoniae pneumonia
RMPP: Refractory mycoplasma pneumoniae pneumonia
VTM: Viral transport medium
HRV: Human rhinovirus
HPIV: Human parainfluenza virus
HMPV: Human metapneumovirus
RSV: Respiratory syncytial virus
HBoV: Human bocavirus
M: Pneumoniae Mycoplasma pneumoniae
C: Pneumoniae Chlamydia pneumoniae
HCoV: Human coronavirus
